# Kinetics and synthesis of poly(3-hydroxybutyrate) by a putative-mutant of *Bacillus licheniformis*

**DOI:** 10.1186/s40643-024-00750-y

**Published:** 2024-04-22

**Authors:** Sikander Ali, Faiza Shabbir Lodhi, M. Usman Ahmad, Qaiser Farid Khan, Asad-ur-Rehman, Abeera Ahmed, Iram Liaqat, M. Nauman Aftab, Tawaf Ali Shah, Ahmad Mohammad Salamatullah, Gezahign Fentahun Wondmie, Mohammed Bourhia

**Affiliations:** 1 Institute of Industrial Biotechnology (IIB), GC University Lahore, Lahore, Pakistan; 2 Department of Zoology, GC University Lahore, Lahore, Pakistan; 3 College of Agriculture Engineering and Food Sciences, Shandong University of Technology, Zibo, 25500 China; 4 Department of Food Science & Nutrition, College of Food and Agricultural Sciences, King Saud University, 11 P.O. Box 2460, Riyadh, 11451 Saudi Arabia; 5 Department of Biology, Bahir Dar University, PO Box 79, Bahir Dar, Ethiopia; 6 Laboratory of Biotechnology and Natural Resources Valorization, Faculty of Sciences, Ibn Zohr University, Agadir, 80060 Morocco

**Keywords:** Kinetics, *Bacillus licheniformis*, Submerged culture, Poly(3-hydroxybutyrate), Fermentation optimizations, Industrial biotechnology

## Abstract

The present study deals with the kinetics of improved poly(3-hydroxybutyrate) (PHB) production by an L-cysteine HCl-depressed mutant of *Bacillus licheniformis.* Production of biodegradable polymers is to eliminate use of materials derived from petrochemicals and also because of their environmental impact. For the current study, mutant strain (NA-21) & wild-type (IIB-isl19) were used for PHB production. Submerged culture with two-stage fermentation technique was used for PHB production. Results indicated that PHB production was improved with 300 mM of –HNO_2_. The superior mutant strain (NA-21) resulted in 2-fold more PHB as compared to the wild-type (IIB-isl9). It was selected, and resistance against L-cysteine HCl was developed. At 4 ppm concentration of L-cysteine HCl, PHB production by mutant strain (NA-cys4) was higher than its wild counterpart by 5.7-fold. Kinetic study of parameters including specific growth rate (µ h^− 1^), growth (Y_x/s_,Y_s/x_), product yield coefficients (Y_p/s_,Y_p/x_), volumetric rate constants (Q_p_, Q_s_, Q_x_) and specific rate constants (q_p_, q_s_, q_x_), were also accomplished. Moreover, Y_p/x_, Q_p_ and q_p_ = µ × Y_p/x_ were found to be very significant as 1.254 ± 0.06 (g/g biomass), 0.134 ± 0.01 (g/l/h) and 0.168 ± 0.01 (g/g/h), respectively. The effect of fatty acids on PHB production highlighted the improvement in PHB production by 1.94-fold. The highest PHB production during the study was 16.35 ± 3.12 g/l which highlighted its significance (*p* ≤ 0.05) and impact on the overall process. The variation in PBH yield between wild-type and mutant *B. licheniformis* is possibly because of induced DNA interstrand thus making unstable thymidine-thymidine dymers. From the results, it was concluded that improved PHB production on industrial scale is fairly possible and it holds the potential to contribute significantly to plastic circularity in the future.

**Supplementary Information:**

The online version contains supplementary material available at 10.1186/s40643-024-00750-y.

## Introduction

Over the years, plastic materials have acquired a great place in our lives due to their desirable properties (Engels et al. 2013). Major raw material for plastic production is petroleum that is a non-renewable source of energy. Their non-degradable nature has urged the world to look for some other means. PHAs are highly popular biodegradable plastics. Among these, poly 3-hydroxybutyrate (PHB) has also become quite popular (Mohd et al. 2022). PHB can be degraded under aerobic, anaerobic and thermophilic conditions. The biosynthesis and biodegradation of PHB is a cyclic process. The biosynthesis pathway of PHB involves three enzymes i.e., β-ketoacyl-CoA thiolase (phbA), acetoacetyl-CoA reductase (phbB) and PHB polymerase (phbC) that catalyze the reaction in successive steps (Nair et al. 2009; Shrivastav et al. 2013). PHB has found its applications in the field of tissue engineering, surgical meshes, wound dressing, cardiovascular and cartilage support and tissue scaffolding for the regeneration of nerves and bones (Kashiwaya et al. 2000; Jose et al. 2022). PHB can be produced from renewable sources i.e., sugars, fatty acids and oils extracted from plants. Soy wastes, malt extracts, hydrolyzed corn starch, waste feed stocks, agro-industrial wastes, whey and extruded rice bran are also potential sources (Wang et al. 2009). Recent studies have indicated the feasibility of producing PHAs using plastic-related substrates as a feedstock (Zhou et al. 2023). It is noteworthy that the process of PHB production holds the potential to contribute significantly to plastic circularity in the future. To stimulate PHB production, a two-stage fermentation technique has been commonly used in submerged fermentation (SmF). The initial stage involves supplying sufficient nutrients for significant biomass increase, while the second stage restricts nutrient availability, promoting PHB accumulation in cellular granules (Patwardhan and Srivastava 2004). It was hypothesized that this method can optimize PHB production but after kinetic analysis.

Some versatile prokaryotic microorganisms viz. *Bacillus spp., Ralstonia eutropha*, and *Halmonas boliviensis*, have the ability to synthesize PHB. Bacillus spp. stand out as one of the most extensively studied PHB producers, favored for its stability of replication, plasmid maintenance, high polymer yield, and adaptability to milder fermentation conditions. In particular, *Bacillus megaterium, B. thuringiensis, B. mycoides* and *B. sphaericus* are more prominent (Getachew and Woldesenbet 2016). However, the PHB potential of *B. lichenisformis* being a GRAS bacterium has not been explored earlier. This is a gram-positive bacterium that is being exploited in industries to produce several enzymes and antibiotics in the past (Rey et al. 2004; Chavan et al. 2021). Therefore, the study to exploit its potential for PHB production could be an ideal future prospective in the field. The development of industrial strains by induced mutagenesis is in practice with a great deal of interest and benefits over the years. In this regard, nitrous acid being a mutagenic agent, was selected as it can induce DNA interstrand to cross-link, thus may alter the DNA structure quite significantly in bacteria (Hu et al. 2023). It was hypothesized that such a study could be useful in determining mechanistic role of -HNO_2_ towards induced mutagenesis which are still poorly established.

In the present study, strain improvement of *B. licheniformis* for PHB production was targeted after exposure with nitrous acid (-HNO_2_). Moreover, to prevent the reversal of mutation, mutant strains were intended to be treated with L-cysteine HCl, as a derepressing agent. The optimization of physical parameters (pH, time of incubation, temperature, size of inoculum) was designed for the best yield of PHB. As, β-oxidation of fatty acids promotes the production of PHB by shifting the TCA cycle to PHB synthesis, therefore, the effect of fatty acids on PHB production was also targeted to gain an insight into the industrial process. Moreover, kinetic studies were carried out which highlighted the potential of *B. licheniformis* for PHB production.

## Materials and methods

The chemicals used in the research work included sodium thioglycolate, L-cysteine HCl, sodium potassium tartarate, sodium hydrogen phosphate, sodium dodecyl sulfate (SDS), crotonic acid, dinitrosalicylic acid (DNS), sodium metabisulfate. These were purchased from Acros (Belgium) and E-Merck (Germany). All these chemicals were of analytical grade, having the maximum possible purity.

### Microorganism and culture maintenance

The culture of *Bacillus licheniformis* (IIB-isl9) was procured from the culture bank of Institute of Industrial Biotechnology (IIB), Government College University Lahore. It was maintained on nutrient agar slants. The cultures were revived after every two weeks and stored at 4 °C. The purity of microbial culture was frequently checked by using standard staining and microscopic procedures after Pal et al. (2009).

### Inoculum preparation

The inoculum was prepared by adding 10 ml sterilized distilled water in two weeks old slant. Bacterial growth on nutrient agar surface was disrupted with a sterile inoculation loop. A bacterial suspension (OD_575nm_∼% 0.6) was prepared by gentle mixing. One milliliter of the bacterial suspension was used to inoculate 50 ml of the production medium (2%, v/v).

### Fermentation technique

Two-stage fermentation technique was followed for PHB production. Multiple fermentation experiments were conducted separately for all the parameters. Fermentation medium for the first stage was glucose minimal medium (pH 7) that contained 8% D-glucose, 0.3% ammonium nitrate, 0.025% potassium dihydrogen phosphate and 0.006% MgSO_4_.7H_2_O. All components were sterilized in an autoclave at 121 °C, 15 psi for 15 min, except D-glucose that was sterilized by syringe filter (0.22 μm). After sterilization, 50 ml of the medium was inoculated with 4% (v/v) inoculum under aseptic conditions. The inoculated flasks were incubated for 72 h in a shaking incubator (VS-8480, Vision Scientific Co. Ltd, Akihabara Japan) at 37 °C and 150 rpm.

After incubation, the medium was centrifuged at 6,000 rpm for 15 min in a centrifuge machine (D-78,532, Hettich Zentrifugen EBA 20, Tuttligen, Germany). The supernatant was discarded, and pellet was collected in 1.5 ml of sterile deionized distilled H_2_O. The collected pellet was inoculated into another medium for the second stage of fermentation. The second medium was nitrogen limited that contained 0.8% nutrient broth and 0.2% (NH_4_)_2_SO_4_. The medium was incubated for 72 h in a shaking incubator at 37 °C and 150 rpm. All the experiments were conducted in a set of three parallel replicates.

### Optimization studies

Various parameters including wavelength (295 nm), pH of fermentation medium (7.5), time of incubation (48 h), temperature (40 °C) and size of inoculum (10%, v/v) were optimized. The individual and combined effect of saturated and un-saturated fatty acids on PHB production was also studied. The addition of lauric acid was found optimal.

### Induced mutagenesis by using –HNO_2_

Mutagenesis was induced in *B. licheniformis* (IIB-isl9) to produce improved PHB by using NA. Different solutions of NA in varying concentrations (50–300 mM) were prepared in acetate buffer (0.02 M, pH 4.5). From each dilution, 2.5 ml was added in 1.5 ml of bacterial suspension (1.2 × 107 CFU/ml, OD_575nm_ ∼ 0.6) of *B. licheniformis* (IIB-isl9) and 1 ml of 0.1 N HCl was also added. The suspension was mixed thoroughly for 10 min. Then, sodium thioglycolate (1 ml, 50 ppm) was used to quench the reaction. The mixture was centrifuged at 4,500 rpm for 20 min. The supernatant was removed, and pellet was washed twice by centrifugation, at the condition mentioned before. About 0.1 ml of washed cells (OD_575nm_ ∼ 0.6) were spread evenly on nutrient agar plates and incubated at 37 °C. A control was also run in parallel. Colonies were observed and screened after 16–24 h for PHB production. Effect of different exposure time of –HNO_2_ (5–30 min) was also evaluated for enhanced PHB production.

### Development of resistance against L-cysteine HCl

Different solutions of L-cysteine HCl were prepared ranging from 2 to 12 ppm. A mixture was made by mixing 2 ml L-cysteine HCl with 2 ml bacterial suspension (OD_575nm_ ∼ 0.6) of mutant strain (NA-cys4). It was incubated for 5 min at 37 °C. The solution was centrifuged at 4,500 rpm for 20 min. The supernatant was removed, and the pellet was washed with acetate buffer (50 mM, pH 3.5). The resistant mutant variants were stored in acetate buffer (2.5 ml) at 4 °C.

### Analytical techniques

#### Biomass development

After incubation, the medium was centrifuged in pre-weighed falcon tubes at 6,000 rpm for 15 min and the supernatant was collected in new falcon tubes for more analysis. Phosphate buffer (50 mM, pH 7) was added to the pellet for washing at 6,000 rpm for 15 min. The washed pellet was dried in a hot air oven at 60 °C for ∼ 20 min. The weight of dried pellet was calculated, and biomass development was calculated in g/l.

### Glucose consumption

DNS test method (after Bailey 1988), was used for the determination of glucose consumption in the broth collected after the centrifugation of nitrogen limited medium. For this, 2 ml of supernatant from each broth and 2 ml of DNS reagent was taken in test tubes. The test tubes were heated in a boiling water bath for 5 min. All test tubes were cooled at room temperature and diluted with distilled water to 10 ml. A blank was also prepared in which 2 ml of broth was replaced by equal volume of distilled water. OD was measured at 545 nm on a spectrophotometer (VIS-1100, Biotechnology Medical Services, Madrid, Spain) and glucose consumption was determined from the standard curve of glucose.

### Extraction and assay of PHB

PHB production was determined in dried cell pellet that was previously used for the determination of biomass development. SDS (5 ml) of 0.5% (w/v) was added to the pellet and vortex to disrupt the pellet. It was incubated at 60 °C for an hour and centrifuged at 6,000 rpm for 30 min. The supernatant was removed and pellet was treated with an extraction mixture of acetone:absolute ethanol:distilled water in 1:1:1 (v/v) for washing and extraction. Finally, boiling chloroform (5 ml) was added to the pellet and evaporated to dryness followed by the addition of 2 ml of conc. H_2_SO_4_ after Law and Slepecky (1961). The solution was heated for 10 min in boiling water bath following its conversion to crotonic acid and cooled at room temperature. Next, the solution was mixed thoroughly and transferred to quartz cuvette for the determination of absorbance at 295 nm wavelength using a UV-spectrophotometer (Genesys 10 S UV-VIS spectrophotometer, Thermo Scientific, London, UK) against concentrated H_2_SO_4_ as blank. The amount of PHB production was determined by the standard curve of crotonic acid.

### Kinetic parameters

Kinetic parameters of fermentation process were determined by the procedures of Pirt and Callow (1959) and Lawford and Roseau (1993). Values for specific growth rate i.e., µ (h^− 1^) were determined from plots of ln(X) vs. time of incubation. Growth yield coefficients (Y_x/s_, Y_s/x_) were determined as the biomass utilized glucose from medium during fermentation. The product yield coefficients (Y_p/s_ and Y_p/x_) were calculated through relationships i.e. Y_p/x_ = dP/dX, Y_p/s_ = dP/dS, respectively.Volumetric rates for product formation (Q_p_), glucose consumption (Q_s_) and biomass development (Q_x_) were calculated from maximum slopes in plots of product formation, glucose consumption and biomass development vs. time of incubation. The specific rate constants for product formation (q_p_), glucose consumption (q_s_) and biomass development (q_x_) were calculated by the multiplication of specific growth rates with growth, and product yield coefficients by the equation i.e. q_p_ = µ × Y_p/x_, q_s_ = µ × Y_s/x_ and q_x_ = µ × Y_x/s_.

### Statistical analysis

Procedure of Snedecor and Cochran (1980) was used to compare the treatment effects. Post hoc multiple comparison tests were applied under one-way ANOVA. Significance was presented in the form of probability (*p* ≤ 0.05) values.

## Results

### Mutagenesis of *B. Licheniformis* wild-type IIB-isl9 and development of resistance against L-cysteine HCl

The effect of different concentrations of -HNO_2_ (50–300 mM) for induced mutagenesis of *B. licheniformis* (wild-type IIB-isl9), and development of resistance against L-cysteine HCl for improved PHB production was studied, as depicted in Table 1. The number of survivor colonies decreased with increasing NA concentration. At 50 mM, the death rate was 70% with 6 survivor colonies, while at 300 mM, the death rate was 90% with only 1 survivor colony. PHB production from the wild type was 0.56 ± 0.12 g/l, but treatment with NA improved PHB production due to increased enzyme activity. The strain NA-21 at 300 mM showed maximum PHB production at 1.12 ± 0.3 g/l twice that of IIB-isl9, making it the preferred choice for further treatments. The effect of different NA exposure times (5–30 min) on NA-21 was evaluated. Longer exposure increased the death rate, suggesting that only mutants capable of withstanding mutation effects survived. The minimum death rate was 80% at 10 min, rising to 95% at 25 min. Survivor colonies decreased with higher NA concentration, with 7 colonies observed initially, 4 after 10 min, and only 1 after 25 min. PHB production improved with longer exposure, reaching a maximum of 3.33 ± 0.51 g/l for NA-t8 at 25 min, making it the selected variant for further treatment.

**Table 1:** NA induced mutagenesis of *B. licheniformis* (wild-type IIB-isl9) and development of resistance against L-cysteine HCl for improved PHB production

Different Treatments	Death rate (%)	No. of survivor colonies	Strain coding	Range of PHB production (g/l)
Wild-type IIB-isl9				0.56 ± 0.12
NA conc. (mM)^$^				
50	70	6	NA-1, NA-2, NA-3, NA-4, NA-5, NA-6	0.03–0.38
100	75	2	NA-7, NA-8	0.31–0.45
150	80	5	NA-9, NA-10, NA-11, NA-12, NA-13	0.46–0.64
200	85	4	NA-14, NA-15, NA-16, NA-17	0.65–0.81
250	85	3	NA-18, NA-19, NA-20	0.82–1.09
300	90	1	NA-21	1.12 ± 0.3
NA exposure time (min)^˄^				
5	80	-	-	-
10	85	4	NA-t1, NA-t2, NA-t3, NA-t4	0.97–1.68
15	90	3	NA-t5, NA-t6, NA-t7	1.08–2.95
20	90	-	-	-
25	95	1	NA-t8	3.22 ± 0.51
30	100	-	-	-
L-Cysteine HCl conc. (ppm)				
2	85	-	-	-
4	98	1	NA-cys4	3.19 ± 0.43
6	100	-	-	-
8	100	-	-	-
10	100	-	-	-
12	100	-	-	-

$The exposure time was 10 min. ˄NA conc. was 300 mM

Time of incubation 72 h, temperature 37 °C, size of inoculum 4% (v/v), pH 7

±Indicate standard deviation (sd set at 5%) amongst the values of three parallel replicates. The sum mean values differ significantly at *p* ≤ 0.05 from each other in one-set, under one-way analysis of variance (ANOVA)

The resistance against L-cysteine HCl (2–12 ppm) was developed in mutant variant NA-t8. Higher L-cysteine HCl concentrations increased the death rate from 85 to 100%. Only one colony (NA-cys4) survived at 4 ppm concentration, showing improved PHB production at 3.19 ± 0.43 g/l. NA-cys4 was selected for kinetic and parametric optimization.

### Evaluation wavelength for PHB production

**Fig. 1:**
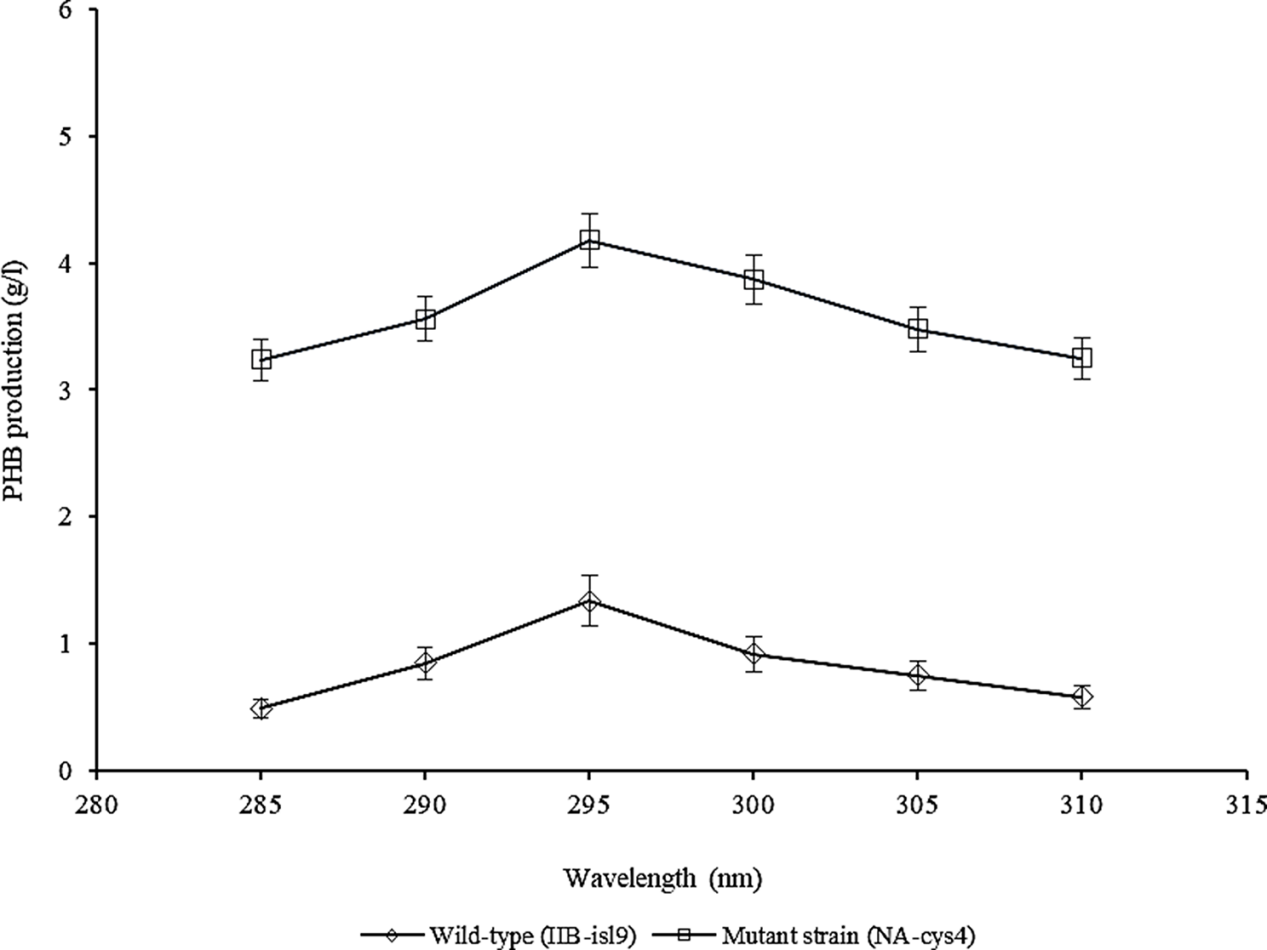
Optimization of wavelength for PHB production by wild-type (IIB-isl9) and mutant strain (NA-cys4) of *B. licheniformis*. Time of incubation 72 h, temperature 37 °C, size of inoculum 4% (v/v), pH 7. Y-error bars indicate standard deviation (sd set at 5%) amongst the values of three parallel replicates. The sum mean values differ significantly at *p* ≤ 0.05 from each other in one-set, under one-way ANOVA

The impact of different wavelengths (ranging from 285 to 310 nm) on PHB production in wild-type (IIB-isl9) and mutant strain (NA-cys4) is depicted in Fig. 1. At 285 nm, IIB-isl9 produced 0.49 ± 0.13 g/l of PHB. PHB production increased as the wavelength was raised, reaching a peak of 1.34 ± 0.07 g/l at 295 nm. However, further increases in wavelength led to a substantial decrease in PHB production. Similarly, NA-cys4 also exhibited increased PHB production with higher wavelengths, reaching a maximum of 4.18 ± 1.02 g/l at 295 nm, which was 3.11 times higher than IIB-isl9. However, at 310 nm, PHB production decreased to 3.25 ± 0.95 g/l.

### Effect of pH on PHB production

**Fig. 2:**
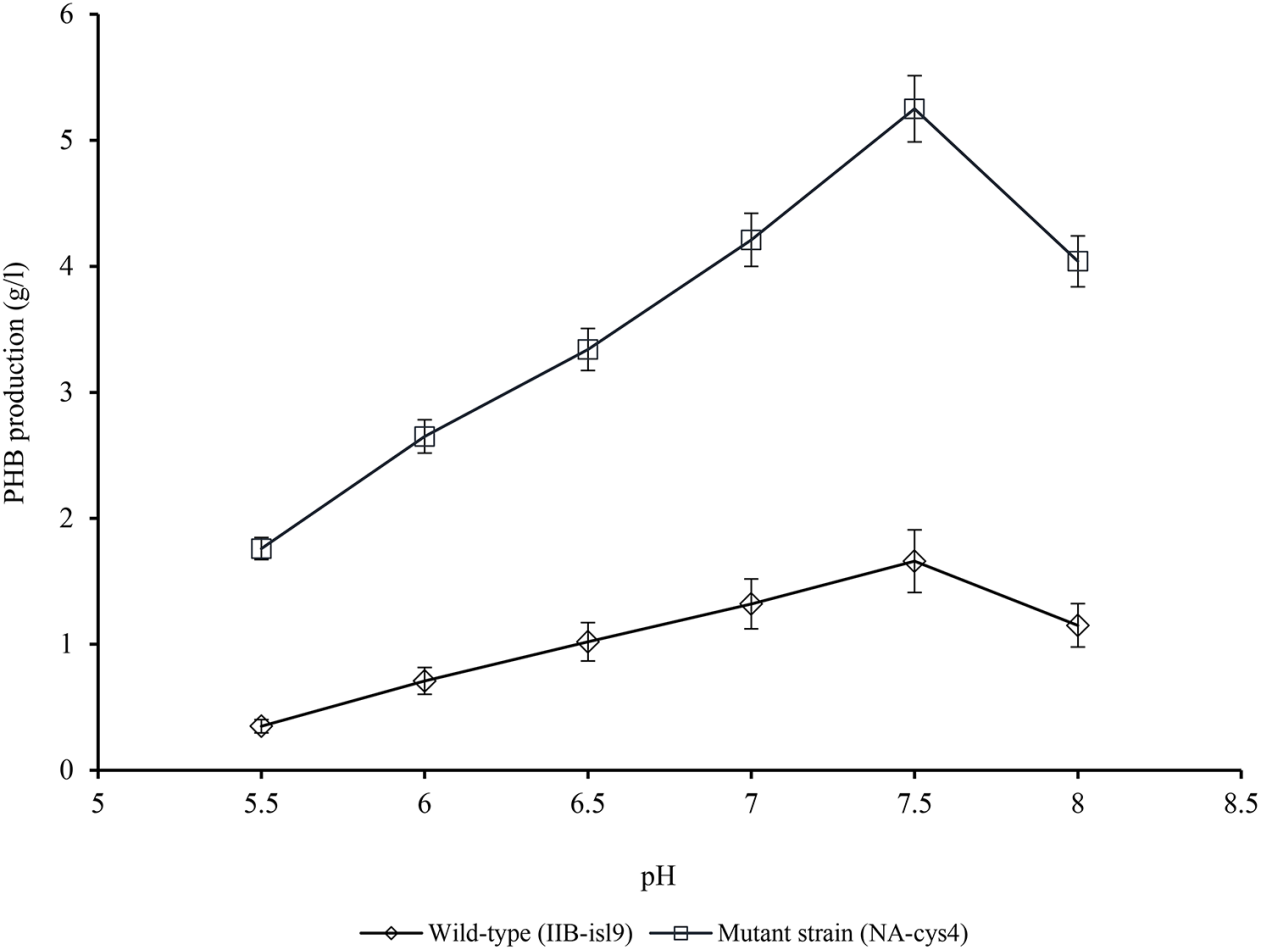
Optimization of pH for PHB production by wild-type (IIB-isl9) and mutant strain (NA-cys4) of *B. licheniformis.* Time of incubation 72 h, temperature 37 °C, size of inoculum 4% (v/v). Y-error bars indicate standard deviation (sd set at 5%) amongst the values of three parallel replicates. The sum mean values differ significantly at *p* ≤ 0.05 from each other in one-set, under one-way ANOVA

The effect of different pH levels (ranging from 5.5 to 8) on PHB production in wild-type (IIB-isl9) and mutant strain (NA-cys4) was investigated (Fig. 2). Both IIB-isl9 and NA-cys4 showed growth and PHB production within the pH range of 5.5-8. At pH 5.5, IIB-isl9 and NA-cys4 produced 0.35 ± 0.09 g/l and 1.76 ± 0.31 g/l of PHB, respectively. PHB production increased with higher pH levels, peaking at pH 7.5, with 1.66 ± 0.44 g/l for IIB-isl9 and 5.25 ± 1.25 g/l for NA-cys4. NA-cys4 exhibited 3.16-fold improved PHB production compared to IIB-isl9. However, further increases in pH resulted in decreased PHB production, with IIB-isl9 producing 1.15 ± 0.28 g/l at pH 8.

### Effect of time incubation on PHB production

**Fig. 3:**
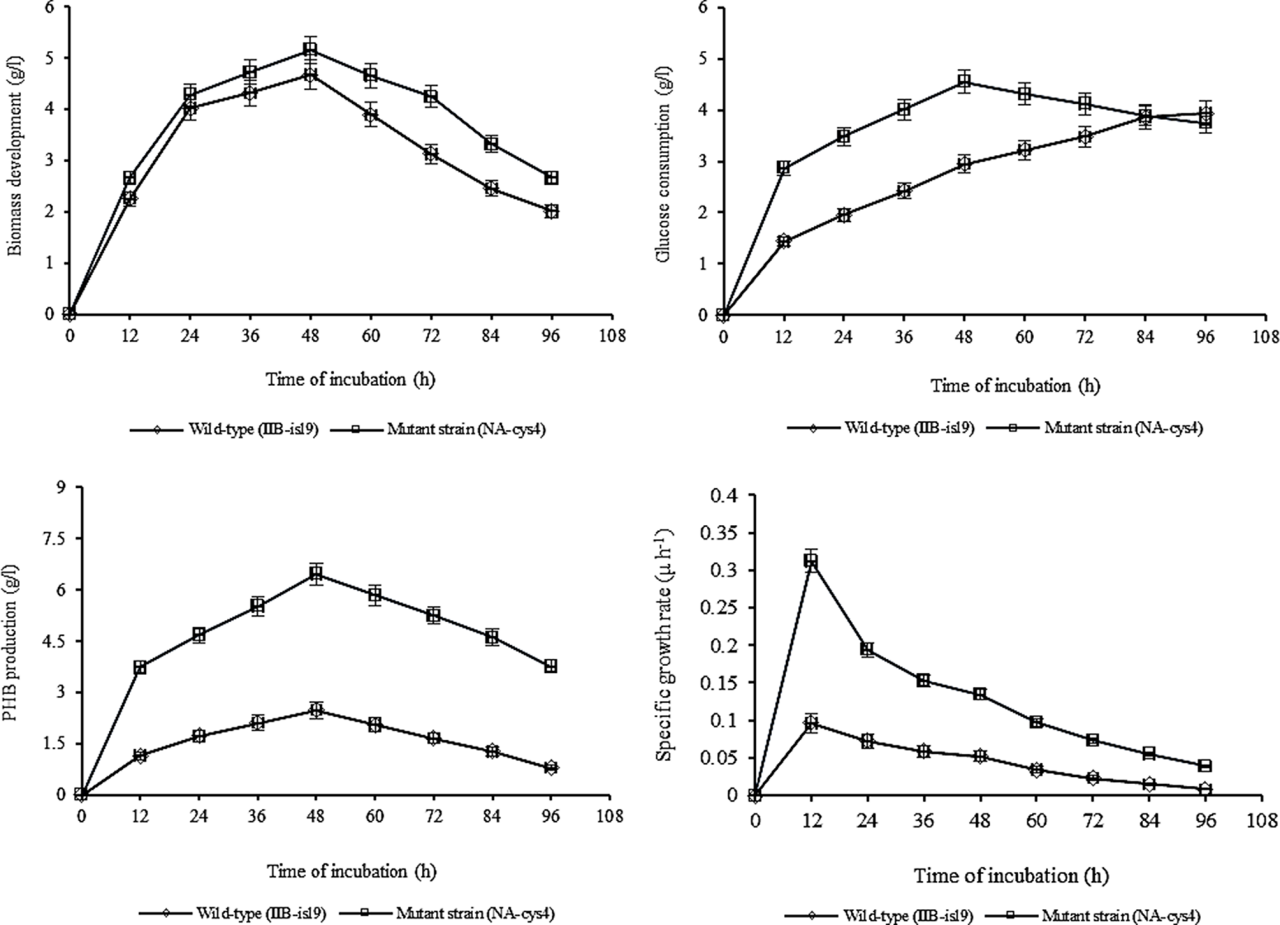
Time of incubation for PHB production by wild-type (IIB-isl9) and mutant strain (NA-cys4) of *B. licheniformis.* Temperature 37 °C, size of inoculum 4% (v/v), pH 7.5. Y-error bars indicate standard deviation (sd set at 5%) amongst the values of three parallel replicates. The sum mean values differ significantly at *p* ≤ 0.05 from each other in one-set, under one-way ANOVA

Figure 3a illustrates the impact of different incubation times on biomass development in wild-type (IIB-isl9) and mutant strain (NA-cys4). At 12 h of incubation, IIB-isl9 and NA-cys4 showed biomass development of 2.25 ± 0.11 g/l and 2.66 ± 0.13 g/l, respectively. As incubation time increased beyond 12 h, biomass development improved significantly. At 48 h, the highest biomass development was observed, reaching 4.67 ± 0.23 g/l for IIB-isl9 and 5.15 ± 0.26 g/l for NA-cys4. However, further prolonging incubation time led to a gradual decrease in biomass development, with IIB-isl9 showing 2.01 ± 0.10 g/l at 96 h.

In Fig. 3b, the effect of different incubation times on glucose consumption is shown. At 12 h of incubation, IIB-isl9 and NA-cys4 exhibited glucose consumption of 1.44 ± 0.07 g/l and 2.87 ± 0.14 g/l, respectively. With prolonged incubation beyond 12 h, glucose consumption increased. The maximum glucose consumption was observed at 48 h, with IIB-isl9 consuming 2.95 ± 0.15 g/l and NA-cys4 consuming 4.55 ± 0.23 g/l. However, when incubation time was extended beyond 48 h, glucose consumption decreased to 3.75 ± 0.19 g/l for NA-cys4. The increase in bacterial population up to 48 h led to higher glucose consumption due to increased biomass. However, after 48 h, glucose consumption declined as the growth rate slowed down.

Figure 3c shows the effect of different incubation times (12–96 h) on PHB production by IIB-isl9 and NA-cys4. PHB production increased with longer incubation times. At 12 h, IIB-isl9 produced 1.15 ± 0.06 g/l of PHB. This production further improved, reaching a peak of 2.48 ± 0.12 g/l at 48 h. Similarly, NA-cys4 exhibited increased PHB production, rising from 3.74 ± 0.19 to 6.46 ± 0.32 g/l at 48 h, which was 2.60 times higher than IIB-isl9 at the same time point. Thus, NA-cys4 showed superior PHB production compared to IIB-isl9. However, beyond 48 h, PHB production gradually decreased, with IIB-isl9 recording 0.79 ± 0.04 g/l at 96 h.

Figure 3d illustrates the impact of different time of incubation on the specific growth rate of IIB-isl9 and NA-cys4. At 12 h of incubation, IIB-isl9 exhibited a specific growth rate of 0.096 ± 0.05 µ h-1, while NA-cys4 showed a higher rate of 0.312 ± 0.02 µ h-1. As the incubation time increased, the specific growth rate decreased for both strains. At 48 h, IIB-isl9 had a specific rate constant of 0.052 ± 0.03 µ h-1, whereas NA-cys4 displayed a significantly higher value of 0.134 ± 0.01 µ h-1, being 2.58-fold greater than IIB-isl9. Further prolonging the incubation time led to a continued decline in the specific growth rate, reaching 0.008 ± 0.01 µ h-1 by IIB-isl9 at 96 h.

### Kinetic study for PHB production

Table 2 presents the impact of incubation time (24–48 h) on kinetic parameters, including growth (Yx/s and Ys/x) and product yield coefficients (Yp/x and Yp/s), specific rate constants (µ, qx, qs, qp = µ × Yp/s, and qp = µ × Yp/x), and volumetric rates (Qp, Qx, and Qs). Yp/x, qp = µ × Yp/x, and Qp are highly significant. Yx/s decreased with longer incubation time, being 1.583 ± 0.08 g biomass/g by IIB-isl9 and 1.132 ± 0.06 g biomass/g by NA-cys4 at 48 h. Conversely, Ys/x increased over time. At 48 h, Ys/x was 0.632 ± 0.03 g/g biomass and 0.883 ± 0.04 g/g biomass for IIB-isl9 and NA-cys4, respectively. NA-cys4 exhibited a 2.36-fold higher Yp/x (1.254 ± 0.06 g/g biomass) compared to IIB-isl9 (0.531 ± 0.03 g/g biomass) at 24 h, which further increased beyond 48 h. In contrast, Yp/s showed a decreasing trend with time, reaching 0.201 ± 0.01 g/g for IIB-isl9 and 1.421 ± 0.07 g/g for NA-cys4 at 48 h. A gradual decrease was observed for IIB-isl9 at 96 h. Specific rate constants and volumetric rates were also studied, with NA-cys4 showing increasing qp = µ × Yp/s and decreasing qp = µ × Yp/x with longer incubation. Qx and Qs decreased with time, with NA-cys4 exhibiting higher values.

**Table 2:** Comparison of different kinetic parameters for PHB production by wild-type (IIB-isl9) and mutant strain (NA-cys4) of *B. licheniformis*

Kinetic parameters	Different incubation periods (h)							
	24		48		72		96	
	IIB-isl9	NA-cys4	IIB-isl9	NA-cys4	IIB-isl9	NA-cys4	IIB-isl9	NA-cys4
Yield coefficients								
Growth yield coefficients								
Y_x/s_ (g biomass/g)	2.062 ± 0.10	1.231 ± 0.06	1.583 ± 0.08	1.132 ± 0.06	0.897 ± 0.04	1.031 ± 0.05	0.511 ± 0.02	0.709 ± 0.04
Y_s/x_ (g/g biomass)	0.485 ± 0.02	0.813 ± 0.04	0.632 ± 0.03	0.883 ± 0.04	1.115 ± 0.06	0.972 ± 0.05	1.961 ± 0.10	1.411 ± 0.07
Product yield coefficients								
Y_p/x_ (g/g biomass)	0.428 ± 0.02	1.091 ± 0.05	0.531 ± 0.03	1.254 ± 0.06	0.532 ± 0.03	1.238 ± 0.06	0.393 ± 0.02	1.411 ± 0.07
Y_p/s_ (g/g)	0.882 ± 0.04	1.341 ± 0.07	0.841 ± 0.04	1.421 ± 0.07	0.477 ± 0.02	1.274 ± 0.06	0.201 ± 0.01	1.001 ± 0.05
Specific rate constants								
Specific rate constants for product formation								
q_p_*^1^ (g/g/h)	0.064 ± 0.03	0.066 ± 0.03	0.044 ± 0.02	0.191 ± 0.01	0.011 ± 0.01	0.093 ± 0.05	0.002 ± 0.01	0.039 ± 0.01
q_p_*^2^ (g/g/h)	0.031 ± 0.02	0.212 ± 0.01	0.028 ± 0.01	0.168 ± 0.01	0.012 ± 0.01	0.091 ± 0.04	0.003 ± 0.01	0.055 ± 0.03
Specific rate constants for biomass development and glucose consumption								
q_x_ (g/g/h)	0.148 ± 0.01	0.239 ± 0.01	0.082 ± 0.04	0.152 ± 0.01	0.021 ± 0.01	0.075 ± 0.03	0.004 ± 0.01	0.028 ± 0.01
q_s_ (g/g/h)	0.035 ± 0.01	0.158 ± 0.01	0.033 ± 0.02	0.118 ± 0.01	0.026 ± 0.01	0.071 ± 0.04	0.016 ± 0.01	0.055 ± 0.03
Volumetric rates								
Volumetric rates for product formation								
Q_p_(g/l/h)	0.072 ± 0.01	0.194 ± 0.01	0.052 ± 0.03	0.134 ± 0.01	0.023 ± 0.01	0.073 ± 0.04	0.008 ± 0.01	0.039 ± 0.01
Volumetric rates for biomass development and glucose consumption								
Q_x_ (g biomass/l/h)	0.168 ± 0.01	0.178 ± 0.01	0.097 ± 0.05	0.107 ± 0.01	0.043 ± 0.02	0.059 ± 0.03	0.021 ± 0.01	0.028 ± 0.01
Q_s_ (g/l/h)	0.081 ± 0.04	0.145 ± 0.01	0.061 ± 0.03	0.095 ± 0.05	0.048 ± 0.02	0.057 ± 0.03	0.041 ± 0.02	0.039 ± 0.02

Yx/s (g biomass/g) = dX/dS, Ys/x (g/g biomass) = dS/dX, Yp/x (g/g biomass) = dP/dX, Yp/s (g/g) = dP/dS. qp (g/g/h) = *1(µ × Yp/s), *2(µ × Yp/x), qx(g/g/h) = µ × Yx/s, qs(g/g/h) = µ × Ys/x. QP (g/l/h) = Slope of product formation, Qx (g biomass/l/h) = Slope of biomass development vs. incubation period and Qs (g/l/h) = Slope of glucose consumption vs. incubation period

µ h-1 = specific growth rate, calculated by plots of ln(X) vs. incubation period

±Indicate standard deviation (sd set at 5%) amongst the values of three parallel replicates. The sum mean values differ significantly at *p* ≤ 0.05 from each other in one-set, under one-way ANOVA

### Effect of temperature on PHB production

**Fig. 4:**
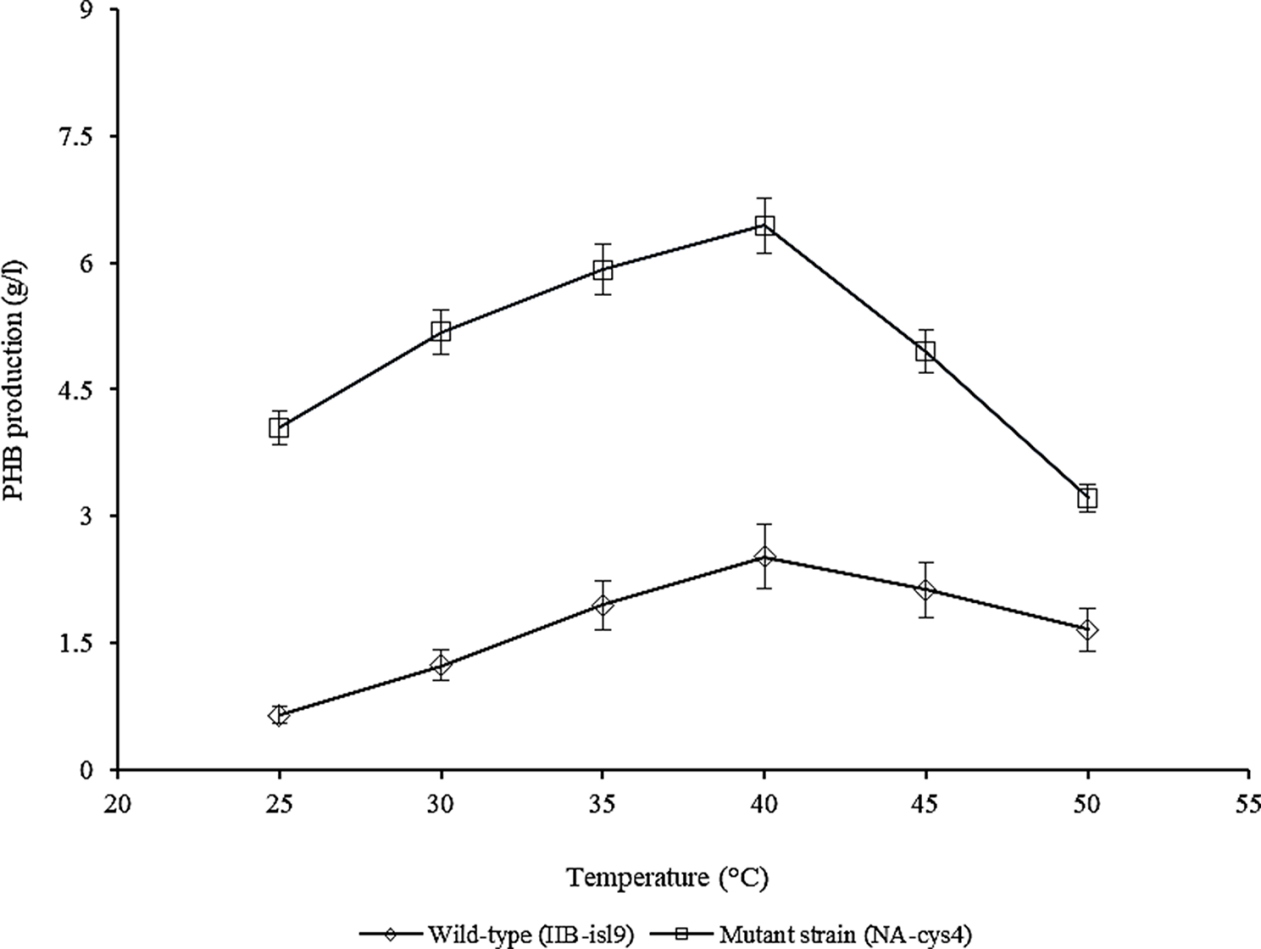
Optimization of temperature for PHB production by wild-type (IIB-isl9) and mutant strain (NA-cys4) of *B. licheniformis.* Time of incubation 48 h, size of inoculum 4% (v/v), pH 7.5. Y-error bars indicate standard deviation (sd set at 5%) amongst the values of three parallel replicates. The sum mean values differ significantly at *p* ≤ 0.05 from each other in one-set, under one-way ANOVA

The effect of different temperatures (25–45 °C) on PHB production by wild-type (IIB-isl9) and mutant strain (NA-cys4) is highlighted in Fig. 4. At 25 °C, IIB-isl9 showed 0.65 ± 0.15 g/l PHB production. Whereas, NA-cys4 produced 4.05 ± 1.05 g/l of PHB. When temperature was increased to 37 °C, PHB production (g/l) was also enhanced. At 37 °C, the maximum PHB production by IIB-isl9 was observed as 2.52 ± 0.64 g/l. Whereas, NA-cys4 showed improved PHB production i.e., 6.44 ± 1.92 g/l at 37 °C that was 2.55-fold higher than that of IIB-isl9. However, by further increase in temperature PHB production was decreased to 1.66 ± 0.32 g/l and 3.22 ± 1.04 g/l by IIB-isl9 and NA-cys4, respectively at 45 °C.

### Effect of size of inoculum on PHB production

**Fig. 5:**
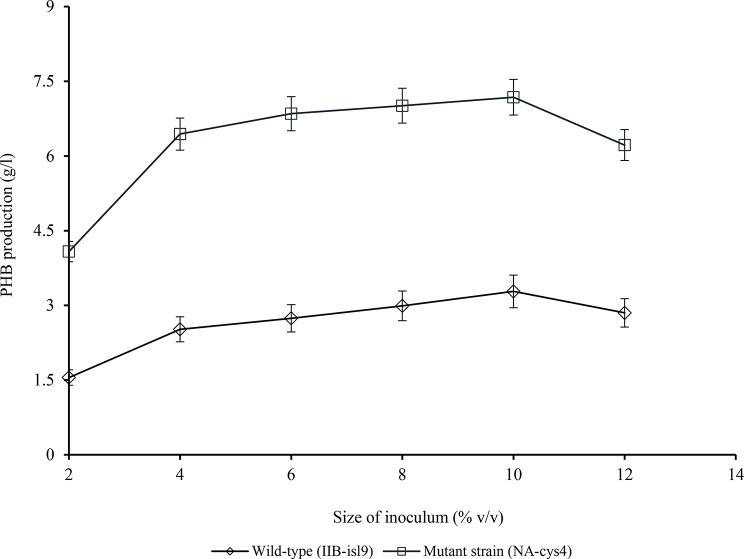
Optimization of size of inoculum for PHB production by wild-type (IIB-isl9) and mutant strain (NA-cys4) of *B. licheniformis.* Time of incubation 48 h, temperature 37 °C, pH 7.5.Y-error bars indicate standard deviation (sd set at 5%) amongst the values of three parallel replicates. The sum mean values differ significantly at *p* ≤ 0.05 from each other in one-set, under one-way ANOVA

The effect of different size of inoculum (2–12% v/v) on PHB production by wild-type (IIB-isl9) and mutant strain (NA-cys4) was studied as shown in Fig. 5. When the size of inoculum was 2% (v/v), PHB production by IIB-isl9 and NA-cys4 was 1.55 ± 0.25 and 4.08 ± 1.02 g/l, respectively. By increasing the size of inoculum, PHB production was also enhanced. At 10% (v/v) it was found to be the maximum i.e., 3.28 ± 1.14 g/l by IIB-isl9 and 7.18 ± 2.02 g/l by NA-cys4. There was 2.19-fold increase in PHB production by NA-cys4 as compared to IIB-isl9, at 10% (v/v).

### Role of saturated and un-saturated fatty acids for PHB production

**Fig. 6:**
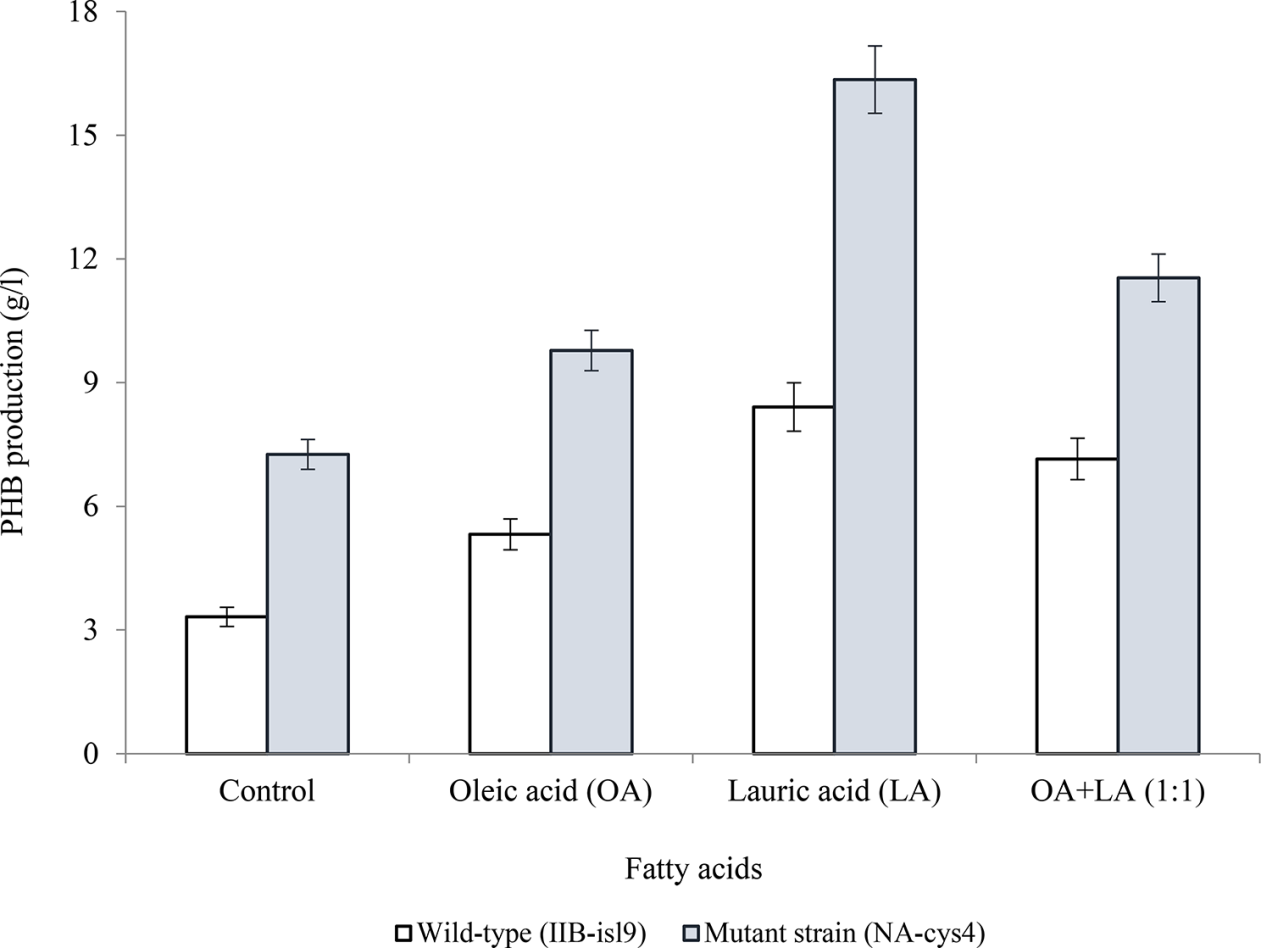
Effect of saturated and un-saturated fatty acids on PHB production by wild-type (IIB-isl9) and mutant strain (NA-cys4) of *B. licheniformis.* Time of incubation 48 h, temperature 37 °C, size of inoculum 10% (v/v), pH 7.5. *The volume of fatty acid was 0.1 ml in each case. Y-error bars indicate standard deviation (sd set at 5%) amongst the values of three parallel replicates. The sum mean values differ significantly at *p* ≤ 0.05 from each other in one set, under one-way ANOVA

In Fig. 6 the role of saturated and un-saturated fatty acids for PHB production is described. PHB production by IIB-isl9 and NA-cys4 was compared when oleic acid (OA), lauric acid (LA) and OA + LA (1:1) were used. A control was also run in parallel. PHB production was increased with fatty acids as compared to the control. PHB production by IIB-isl9 and NA-cys4 was found to be least i.e., 5.32 ± 1.13 and 9.78 ± 2.23 g/l with OA. Whereas LA provided the highest PHB production of 8.41 ± 2.01and 16.35 ± 3.12 g/l by IIB-isl9 and NA-cys4, respectively.

## Discussion

PHB is a biodegradable polymer naturally present in microorganisms. It serves as a reserve for both carbon and energy, however, it has limited carbon storage capacity. Under physiological stress, PHB is synthesized in the cytoplasm. However, with increased PHB concentration, the organism can better withstand high temperatures and oxidative stress. *Bacillus licheniformis* has the ability to synthesize PHB. This bacterium is known to be one of the well performing microorganisms with PHB production rate of over 50%. The aim of this study was to enhance the production of PHA (including PHB) by modifying the genetic composition of *Bacillus licheniformis in* a systematic manner.

Nitrous acid [HNO_2_] is a known mutagen that causes the deamination of DNA bases as reported by Siddiqi (1962). Exposure of bacterial suspension to higher concentrations of NA increased the death rate, likely due to adverse mutation effects (Table 1). Azin and Noroozi (2001) also reported increased enzyme activity with NA mutagenesis in *Aspergillus oryzae* PTCC 5164. Whereas, Ali and Nawaz (2017) also observed resistance development in *Aspergillus oryzae* mutant with low L-cysteine HCl concentration.

The effect of wavelength on PHB production was studied. At 310 nm PHB production decreased (Fig. 1). This observation suggests that the accurate determination of solution concentration relies on selecting the specific wavelength where the solution’s absorbance is at its maximum (Mantele and Deniz 2017). In a different study, Getachew and Woldesenbet (2016) carried out the determination of PHB in a range of 200–320 nm and reported the maximum concentration of PHB at 240 nm. Similarly, Thammasittirong et al. (2017) used 235 nm wavelength for PHB determination.

The pH variations can influence enzyme-substrate binding due to ionization effects (Gray et al. 2010). Increase in pH resulted in decreased PHB production (Fig. 2). The observed decrease may be attributed to changes in membrane transport, enzymatic processes, and enzyme activity when the medium’s pH deviates from its optimum level (Vijayalakshmi et al. 2013). As a result, decrease in growth and PHB production was observed. In a similar study, Ansari and Fatima (2016) also reported the pH of 7.5 as the optimal pH for PHB production. These results are in line with Vijayalakshmi et al. (2013) too, who reported the pH range of 6-7.5 for growth and PHB production. However, in another study, Getachew and Woldesenbet (2016) reported pH 7 for the highest PHB production.

Prolonged incubation resulted in decreased biomass. This decline might be attributed to entering the stationary phase after 48 h, during which growth ceases (Fig. 3a). Further elongation of incubation time resulted in the death phase, where bacteria likely faced competition or were affected by toxins produced by other bacteria (Engels et al. 2013). Longer incubation periods resulted in the decreased glucose consumption (Fig. 3b). Engels et al. 2013 also reported decrease in sugar consumption at higher incubation periods. Incubation time effects PHB production. In present study, beyond 48 h incubation PHB production gradually decreased (Fig. 3c). These results are in complete agreement with (Rohini et al. 2006; Thammasittirong et al. 2017). This might be due to the reason that when the bacteria were in their log phase, exponential increase in growth and PHB accumulation occurred. But when the time of incubation was prolonged further, PHB production was reduced might be due to the lack of micronutrients and accumulation of metabolites that had negative impact on PHB production (Flora et al. 2010). Moreover, medium viscosity might be increased after 48 h of incubation, which resulted into the limitation of air supply (Stam et al. 1986). Therefore, bacteria started to consume stored PHB as energy reserve and a fall in PHB production was observed. Decreased growth rate was observed in prolonged incubation. Similar findings were reported by Pirt and Callow (1959) and Lawford and Roseau (1993).

Impact of incubation period on kinetic parameters such as growth, product yield coefficients, specific rate constants, and volumetric rates were studied (Table 2). Longer incubation times resulted in decrease in kinetic parameters after 96 h. Similar observations were also reported by Yeo et al. (2018) who used xylose and glucose as substrates for the under considered biochemical process.

The effect of different temperatures (25–45 °C) on PHB production by wild-type (IIB-isl9) and mutant strain (NA-cys4) was studied (Fig. 4). Increase in temperature resulted in decreased PHB production. This might be due to the reason that at optimum temperature, the activity of enzymes was maximum which resulted into the highest product formation. But, at lower temperature, the transport of substrate across the membranes was suppressed which caused reduced PHB production. On the other hand, at higher temperature thermal denaturation of the enzymes in PHB biosynthesis pathway occurred. Getachew and Woldesenbet (2016) also reported 37 °C as the optimum temperature for PHB production. These results suggested that the culture was purely mesophilic. However, in a different study, Ansari and Fatima (2016) have reported 30 °C as the best temperature for PHB production.

The effect of different inoculum size (2–12% v/v) on PHB production by wild-type (IIB-isl9) and mutant strain (NA-cys4) was evaluated in Fig. 5. Increase in inoculum size resulted in increased PHB production. As suggested by Ramadas et al. (2010), who has reported 4% size of inoculum for the maximum PHB production. When the size of inoculum was small, the number of bacteria was quite low. As a result, PHB production was low. But when the size of inoculum was increased, there were more bacteria that produced more PHB. However, when the size of inoculum was increased further, bacteria started to compete for nutrients. So PHB production was reduced due to decrease in the number of PHB producing bacteria. Moreover, bacteria started to utilize stored PHB as energy reserve.

The role of saturated and un-saturated fatty acids for PHB production was studied (Fig. 6). Lauric acid (LA) resulted in higher concentration of PHB. It might be due to the production of different PHAs with short and medium chain length. Short chain PHAs have been identified as the precursors of PHB. Saturated fatty acids addition has been reported to produce short chain PHAs and ultimately PHB by Tripathi and Srivastava (2013). These results were in complete agreement with (Lee et al. 1995) who reported enhanced PHB production with fatty acids.

The optimization of physical parameters and kinetic studies resulted in highly encouraging results, with the highest PHB production reaching to 16.35 ± 3.12 g/l (29.2-fold increase compared to the wild-type). Therefore, the study demonstrated highly significant results (HS, *p* ≤ 0.05) indicating commercial ability of the mutant strain after scale up studies.

## Conclusion

In the present study, PHB production was enhanced by strain improvement of *B. licheniformis* using –HNO_2_ at different concentrations. Mutant strain (NA-21) showed improved PHB production as compared to the wild-type (IIB-isl9) and was selected for resistance against different concentrations of L-cysteine HCl. At 4 ppm concentration of L-cysteine HCl, only one mutant colony survived (NA-cys4). It produced 5.7-fold more PHB as compared to its wild-counterpart. The optimization of physical parameters and kinetic studies resulted in encouraging results, with the highest PHB production reaching to 16.35 ± 3.12 g/l (29.2-fold increase compared to the wild-type). Increase in inoculum size resulted in high PHB production. However, increase in temperature showed decrease in PHB production. Also, longer incubation time revealed (96 h) decrease in the values of kinetic parameters. The results fairly support the idea that the difference in PHB yield between the wild-type and mutant strain of *B. licheniformis* could be correlated to the possibility of induced DNA interstrand to cross-link with each other viz-a-viz making unstable thymidine-thymidine dimers. Overall, the study demonstrated highly significant (HS, *p* ≤ 0.05) results indicating potential ability of the putative mutant strain. However, scale up studies prior to PHB characterization could be a desirable future prospect to exploit the process commercially.

### Electronic supplementary material

Below is the link to the electronic supplementary material.

Supplementary Material 1

## Data Availability

The sequence data of the wild strain has been submitted to NCBI, and the accession number is [PP077077].
