# Supplementary figures and images for: Kinetics and synthesis of poly(3-hydroxybutyrate) by a putative-mutant of Bacillus licheniformis
Source: Bioresour Bioprocess. 2024 Apr 22;11(1):41. doi: 10.1186/s40643-024-00750-y (PMC11033250; doi:10.1186/s40643-024-00750-y)

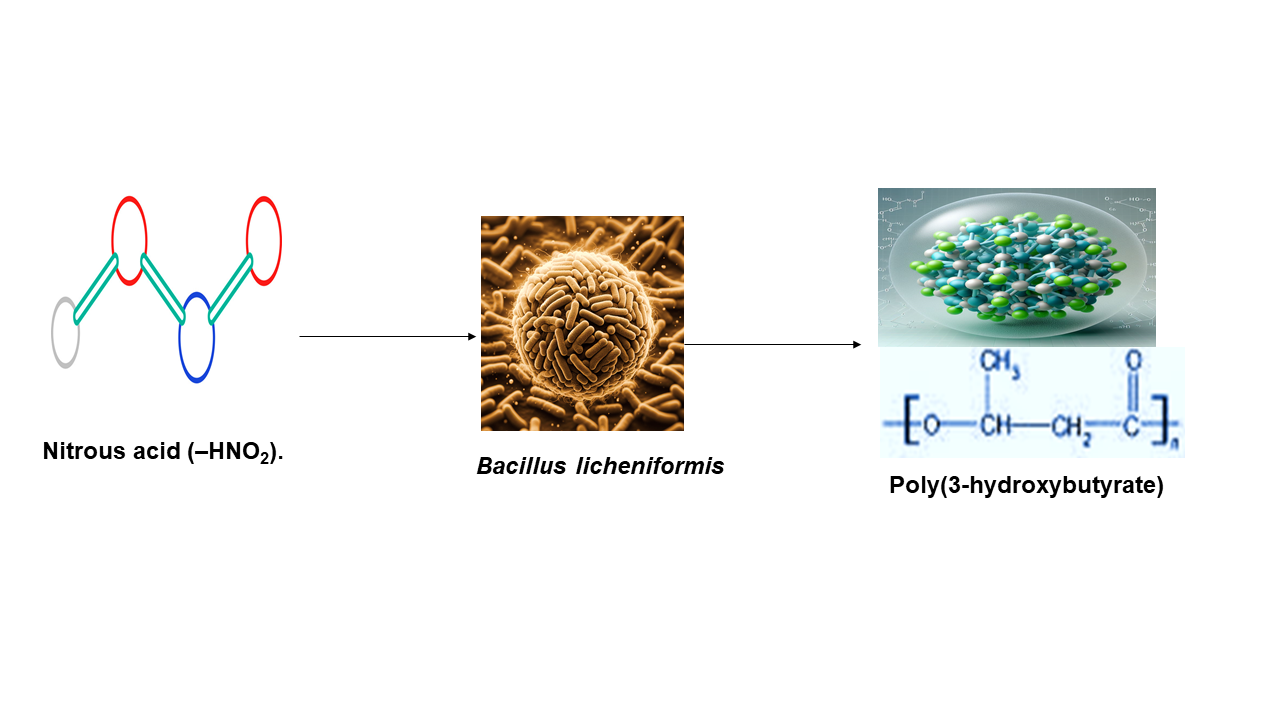

Supplement: Supplementary file 1 — Supplementary Material 1 [file 40643_2024_750_MOESM1_ESM.png]
